# Effect of a Single Dose of Deflazacort on Postoperative Pain, Swelling, and Trismus after Impacted Lower Third Molar Surgery: Randomised Clinical Trial

**DOI:** 10.3390/medicina60081206

**Published:** 2024-07-25

**Authors:** Volkan Kaplan, Levent Ciğerim, Erkan Feslihan, Saadet Çınarsoy Ciğerim

**Affiliations:** 1 Department of Oral and Maxillofacial Surgery, Faculty of Dentistry, Tekirdag Namik Kemal University, Tekirdag 59030, Turkey; vkaplan@nku.edu.tr; 2 Department of Oral and Maxillofacial Surgery, Faculty of Dentistry, Van Yuzuncu Yil University, Van 65080, Turkey; leventcigerim@yyu.edu.tr; 3 Department of Orthodontics, Faculty of Dentistry, Van Yuzuncu Yil University, Van 65080, Turkey; saadetcinarsoy@yyu.edu.tr

**Keywords:** deflazacort, corticosteroid, pain, swelling, trismus, impacted third molar surgery

## Abstract

*Background and Objectives*: The aim of this study was to investigate the efficacy of a single preoperative dose of deflazacort on pain, swelling, and trismus after impacted lower third molar surgery. *Materials and Methods*: This randomised, prospective, double-blind, split-mouth clinical study included 26 healthy individuals with bilaterally impacted lower third molars. Group 1 was given a placebo (single-dose vitamin C tablet), and group 2 was given a single 30 mg dose of deflazacort 1 h prior to surgery. Pain was evaluated using the visual analogue scale for 1 week postoperatively. Oedema (in mm) and trismus (in mm) were evaluated preoperatively and on postoperative days 2 and 7. The Mann–Whitney *U* test was applied for group analyses. *p* values < 0.05 were considered statistically significant. *Results*: Postoperative pain scores were significantly lower in the deflazacort group at the 6th and 12th hours after surgery (*p* < 0.05). There were no significant differences in trismus between the groups at any time point (*p* > 0.05). There was less oedema in the deflazacort group on postoperative days 2 and 7, without any statistically significant difference (*p* > 0.05). *Conclusions*: A single preoperative dose of 30 mg deflazacort was found to be clinically effective in reducing pain and oedema after extraction of impacted lower third molars.

## 1. Introduction

Impacted lower third molars are the most commonly impacted teeth in the jaws, and their treatment usually requires surgical removal. The surgical extraction of these teeth is an important part of the routine workflow of oral and maxillofacial surgery clinics [1,2]. This procedure is associated with several postoperative sequelae that have both social and biological effects. In addition to serious complications such as paraesthesia, infection, jaw fractures, and alveolitis, patients generally complain of pain, oedema, and trismus due to postoperative inflammatory responses [3,4]. The peak intensity of these complications occurs during the first 48 h after surgery, gradually decreasing in severity and resolving within 7 days. Inflammation resulting from surgical trauma is a major contributor to these complications. The overall aim of postoperative interventions is to prevent or reduce complications that affect the patient’s quality of life. There are several treatments and methods for controlling the extent of postoperative complications [5,6].

The treatment methods used to prevent complications after impacted wisdom teeth surgery have been examined, and a number of analgesics have been used to manage postoperative pain and swelling. Among these, paracetamol and NSAIDs stand out as commonly used options, and are often considered essential by many practitioners. In addition, they may be supplemented with opioids or corticosteroids to increase their effectiveness [7,8]. Topical gels containing antimicrobial agents enable the direct application of the drug to postoperative surgical sites. Their efficacy surpasses that of mouthwash due to the gel’s ability to prolong drug release, provide targeted alveolar action, and enhance bioavailability. Unlike mouthwash, which carries a risk of clot dissolution within the first 24 h, gels can be applied immediately after tooth extraction [6,9]. Cryotherapy, which involves applying ice to the area outside the surgical site, is a straightforward method that many clinicians prefer. The principle behind cryotherapy is that lower temperatures induce vasoconstriction, reducing postoperative swelling. In addition, it can reduce nerve fibre conduction velocity, resulting in an analgesic effect [10]. The surgical principles and instruments utilized throughout impacted wisdom teeth surgery have been subjected to investigation for managing healing complications. Envelope flap design and polybutester sutures have been demonstrated to reduce postoperative complications [11,12]. In the context of third molar surgery, irrigation is employed during the postoperative phase to manage alveolar osteitis. The removal of necrotic debris or food particles via irrigation is hypothesized to eliminate potential sources of inflammation and pain [6,13]. Moreover, when extraction sockets are irrigated with rifamycin, there is evidence suggesting a reduction in the incidence of alveolitis, the prevention of infection, and the induction of analgesia following the surgical removal of impacted third molars [13,14].

For more than 50 years, corticosteroids have been used to prevent postoperative complications after impacted lower wisdom tooth surgery, and there are several studies published in the literature regarding this issue [15,16]. The anti-inflammatory effects of corticosteroids are attributed to the inhibition of phospholipase A2, which reduces the release of arachidonic acid derivates. This process leads to a decrease in the synthesis of leukotrienes and prostaglandins, thus preventing the accumulation of neutrophils in the inflamed area [17]. Corticosteroids have two main forms: glucocorticoids and mineralocorticoids. Glucocorticoids, such as endogenous cortisol, primarily demonstrate anti-inflammatory and immunosuppressive effects. These actions entail the inhibition of the production and function of various inflammatory cells and provoke the redistribution of immune cells to different body compartments, resulting in a reduced number of circulating immune cells overall. On the other hand, mineralocorticoids, including endogenous aldosterone, play a role in regulating the balance of salt and water in the body. Specifically, mineralocorticoids act on renal tubules, promoting the reabsorption of sodium and the excretion of potassium [18]. Glucocorticoids, such as dexamethasone, methylprednisolone, and triamcinolone, are commonly used in oral surgery because of their anti-inflammatory activities alongside their minimal effects on fluid and electrolyte balance [19].

Deflazacort belongs to the group of synthetic corticosteroids and is quickly and completely absorbed in the intestinal tract when taken orally [20]. Deflazacort is an oxazoline derivative of prednisolone. It is used for a wide number of different clinical conditions, given its anti-inflammatory and immunosuppressive effects [21,22]. Studies have shown that deflazacort is as effective as prednisone or methylprednisolone in the treatment of rheumatoid arthritis, severe asthma, nephrotic syndrome, Duchenne muscular dystrophy, and systemic lupus erythematosus [23,24]. Compared with other glucocorticoids, deflazacort appears to have limited adverse effects on bone and carbohydrate metabolism at doses with equivalent clinical efficacy [25]. The incidence of gastrointestinal disorders is also lower in patients treated with deflazacort [22,23].

The hypothesis of this study was that a single preoperative dose of deflazacort might be effective in preventing postoperative complications in patients undergoing impacted lower third molar surgery. The purpose of the study was to investigate the efficacy of a single preoperative dose of deflazacort on pain, swelling, and trismus following impacted lower third molar surgery.

## 2. Materials and Methods

This randomised, prospective, double-blind, split-mouth clinical trial was conducted at the Department of Oral and Maxillofacial Surgery, Faculty of Dentistry, Van Yuzuncu Yil University, between September 2017 and September 2018. Ethical approval was confirmed by the Van Yuzuncu Yil University’s Clinical Investigations Ethics Committee, Faculty of Medicine (decision number: 19.07.2017-06, 19 July 2017). The researchers adhered to the Declaration of Helsinki throughout the study. The clinical trial was recorded on ClinicalTrials.gov (registration number: NCT04365088). Written informed consent was obtained from all volunteers after they were informed about the study.

### 2.1. Study Sample

The study included healthy individuals (ASA-1, according to the classification of the American Society of Anaesthesiologists) without any congenital or acquired systemic disorders. Patients were included if they were aged between 18 and 40 years, had bilateral impacted mandibular third molars indicated for surgical extraction due to orthodontic purposes at similar angulations (mesioangular according to Winter’s classification), and had similar degrees of impaction (class II and class B according to the Pell–Gregory classification). Patients with any systemic disease that could interfere with surgical treatment, patients who were smokers, substance abusers, and alcohol drinkers, pregnant or breastfeeding mothers, patients who had taken anti-inflammatory medication within one week before the surgery, and those who had facial asymmetry were excluded from the study (Table 1). In total, 30 patients met the inclusion criteria, but 4 patients were excluded from the study because they failed to attend one or more follow-up visits during the study.

### 2.2. Outcome Variables

Self-reported pain scores during the first postoperative week and mouth opening and facial swelling measurements (in mm) on postoperative days 2 and 7 were used as outcome variables. Surgical interventions and postoperative patient assessments were performed by different researchers.

### 2.3. Patient Allocation

In the trial, patients received a placebo or deflazacort 1 h prior to surgery. Since this was a split-mouth study, each group consisted of 26 patients. Group 1 was given a placebo (single-dose vitamin C tablet), and group 2 was given a single 30 mg dose of deflazacort. A permutation method was used in the MedCalc 11.5.1 software package to randomise which drug would be used on which side and which side would be extracted for the first operation. The surgical team was blinded to group allocation during surgery and the postoperative follow-up period.

### 2.4. Surgical Procedure

The operations were performed by the same surgeons on Monday, Tuesday, and Wednesday mornings according to a standard protocol. The time intervals between the surgical extractions of bilaterally impacted lower third molars were at least 3 weeks. Patients were given 2 mL of articaine hydrochloride local anaesthesia with an epinephrine solution (40 mg/mL; 0.01 mg/mL; Maxicaine Fort; Vem Pharmaceuticals Ltd., Istanbul, Turkey). After the induction of anaesthesia, a three-cornered flap incision was made using a no. 15 scalpel blade, and the mucoperiosteal flap was elevated. The buccal bone was removed, and if necessary, the tooth was divided under physiological saline irrigation using a surgical micromotor running at 1400 rpm and a steel round drill that was 1.6 mm in diameter. After extraction, any granulation tissue was removed, and the extraction socket was irrigated. Once haemostasis was achieved, the flap closure was performed with 3/0 silk sutures. An antibiotic (amoxicillin 875 mg + clavulanic acid 125 mg, twice a day; Augmentin-BID 1000 mg; GlaxoSmithKline Pharmaceuticals Ltd., Istanbul, Turkey), analgesic (600 mg Ibuprofen, twice daily; Brufen 600 mg; Abbott Laboratories Ltd., Istanbul, Turkey), and oral antiseptics (% 0.3 benzydamine hydrochloride, 3 times a day; Tanflex Fort 15 mL spray; Abdi İbrahim Pharmaceuticals Industry and Trade Inc., Istanbul, Turkey) were prescribed as postoperative medication. Antibiotics and analgesics were given immediately after surgery, and mouthwash was started one day after surgery and continued for one week postoperatively. For the first 24 h after surgery, patients were advised to eat only soft foods and not to use dental floss, toothbrushes, or mouthwash.

### 2.5. Data Collection

**Figure 1:**
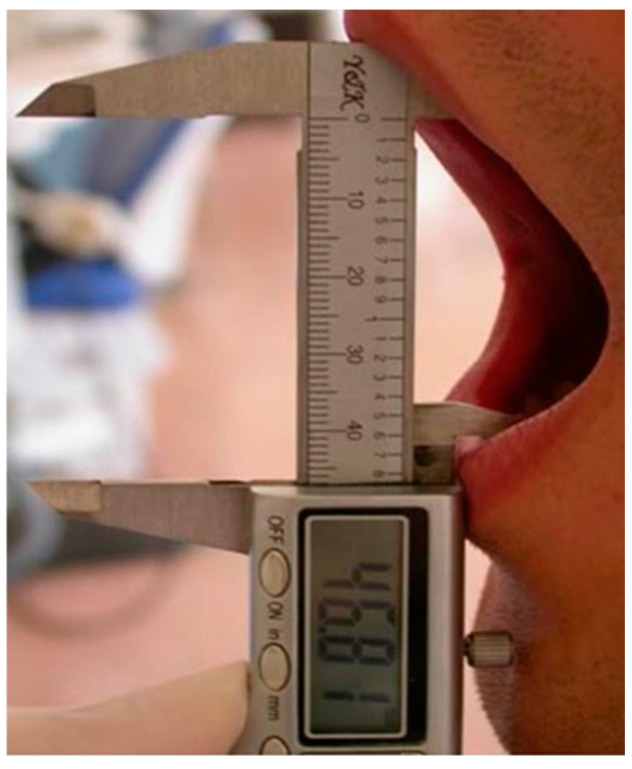
Measurement of Mouth Opening.

The maximum mouth opening (MMO) was measured in millimetres from the incisal edge of the right upper and lower incisors, using a calliper to evaluate the trismus (Figure 1). The preoperative MMO values were subtracted from the MMO values on postoperative days 2 and 7, and the proportional changes in measurements compared to the preoperative MMO were calculated.

**Figure 2:**
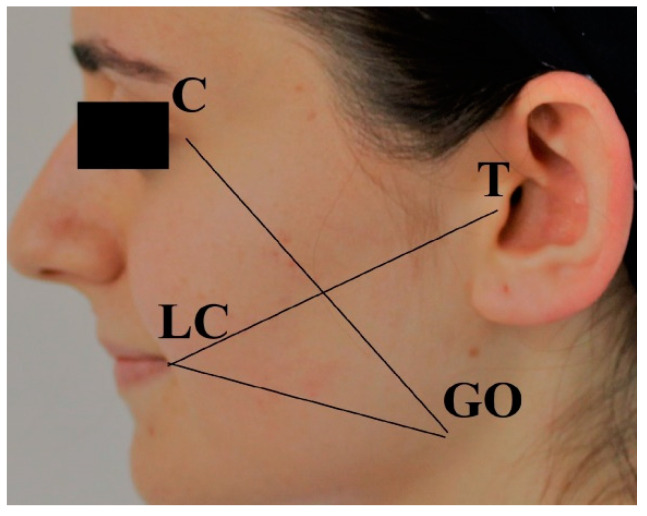
Facial landmarks for assessment of oedema.

The swelling was assessed by measuring the distance between several facial landmarks using a millimetre ruler. Measurement I was taken from the tragus (T) to the labial commissure (LC), measurement II from the labial commissure (LC) to the gonion (Go), and measurement III from the gonion (Go) to the lateral canthus (C) (Figure 2). To evaluate swelling, the preoperative measurements were subtracted from the measurements on postoperative days 2 and 7, and the proportional changes compared to the preoperative measurements were calculated. For each evaluation interval, a single swelling value was obtained by averaging the three measurements, and evaluations were performed according to this single value.

**Figure 3:**
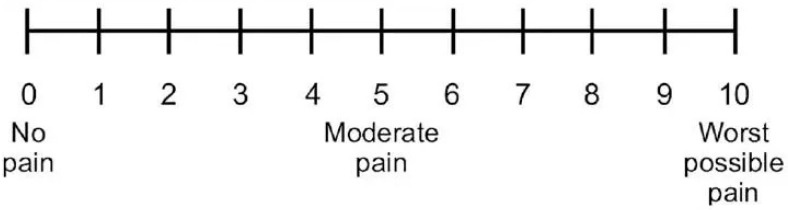
VAS Scale for assessment of postoperative pain.

Patients were given a visual analogue scale (VAS) to rate their postoperative pain at postoperative hours 6, 12, and 24 and on a daily basis from the 2nd to 7th postoperative days (Figure 3). Patients were asked to rate their pain on a 10-point VAS, with 0 being no pain at all and 10 being the worst pain they have experienced until now. On postoperative days 2 and 7, patients were assessed for swelling and trismus. Any complications such as alveolitis and infection were also recorded.

### 2.6. Statistical Analysis

The power analysis was carried out using the PASS programme according to previous studies [26,27], which showed that the standard deviation (SD) for pain scores varied from 4.87 to 5.39. The SD was therefore set at 5. The effect size was assumed by the researcher to be 2 at the 95% confidence level and based on the approximate power of 80%, and a Z value of 1.96 was used for the type I error rate of 0.05. Based on the calculation of the sample size, the sample size was determined to be 24. Taking into account possible losses, a decision was made to include 30 people, which was 25% more than this number. Descriptive statistics (minimum and maximum, standard deviation, mean, and frequency) were calculated for every parameter. The Mann–Whitney *U* test was applied for group analyses. All analyses were performed using SPSS 21.0. *p* values < 0.05 were considered statistically significant.

## 3. Results

Data obtained from 26 patients were analysed. The patients included in the study were 14 females (53.8%) and 12 males (46.2%), and their mean age was 23.31 ± 5.01, ranging from 18 to 35 years (Table 2).

When postoperative pain scores were evaluated, a significant difference was found between the groups only at the 6th and 12th hours (*p* < 0.05). Postoperative pain scores were significantly lower in the deflazacort group (Group 2) at postoperative hours 6 and 12 (*p* < 0.05). However, although there was no statistically significant difference in postoperative swelling between the groups on postoperative day 2, postoperative swelling was experienced less in the second group. In conclusion, the lower swelling values in the first 12 h may have been a factor in the measurement of lower postoperative pain scores in the second group. In terms of further measurements, pain scores were similar until the 5th day (*p* > 0.05) (Table 3).

Even though high trismus values were recorded on the 2nd day, it was observed that trismus was resolved on the 7th day. There were no significant differences in trismus between the groups at any time point (*p* > 0.05) (Table 4).

Facial oedema reaches its maximum level at 48 h hours after surgery and resolves within 7 days. For this reason, the assessments of facial swelling were performed on postoperative days 2 and 7. The evaluation of swelling showed that there was no statistically significant difference between the study and control groups at any time point (*p* > 0.05). Although the time taken for reduction in swelling was similar between the study and control groups, less swelling was observed in the deflazacort group on postoperative days 2 and 7, but without any statistically significant difference (*p* > 0.05) (Table 5). No side effects were observed in either the study or control groups.

## 4. Discussion

The anti-inflammatory activity of deflazacort and other synthetic corticosteroids is the first reason for their therapeutic use. The synthesis of novel compounds with greater anti-inflammatory activity and a reduced incidence of side effects compared to cortisone were the focus of early corticosteroid research. Prednisolone and prednisone, which were synthesized for this purpose, have greater anti-inflammatory activity compared with natural steroids, while mineralocorticoid activity was attenuated by about half [28]. There are other corticosteroids, including fluorinated glucocorticoids such as dexamethasone, which have no mineralocorticoid activity, and some typical adverse events due to their long-term use have been reported. The reality is that the side effects of corticosteroids often affect patients’ quality of life, and concerns about the safety of these drugs are particularly important for certain groups of patients treated with steroids [29,30]. The primary goal of deflazacort synthesis was to create a novel agent that would be more tolerable for all age groups. The unique pharmacological properties of deflazacort are mainly due to its structural design. These properties include a significant reduction in sodium retention, potent immunosuppressive and anti-inflammatory effects, and reduced interference with the metabolism of phosphorus and carbohydrates compared with previous corticosteroids. There are numerous and significant risks relating to adverse effects and toxicities associated with chronic treatment with systemic corticosteroids [31,32]. These drugs affect every organ system and metabolic process in the human body. The risk of adverse effects associated with corticosteroid therapy depends on the dose and duration of the therapy, as well as the specific corticosteroid used [28]. Additionally, in cases of steroid use for less than three weeks, the dose can be stopped suddenly. Therefore, there was no harm in discontinuing the single dose of deflazacort administered to patients [33]. In this study, the authors preferred to use a single dose of deflazacort, which is a corticosteroid for which efficacy has not been evaluated, in a split-mouth study for lower impacted third molar surgery in order to protect patients from the potential side effects of long-term steroid use as described in the literature.

One of the main advantages of deflazacort is that it has fewer side effects on bone metabolism than other corticosteroids. In rheumatoid arthritis, deflazacort may provide protection against bone destruction and joint damage [34]. Deflazacort has been shown to inhibit synovial cell invasiveness and proliferation in a dose-dependent manner by differentially modulating the individual components of the fibrinolytic system. It has been shown in the pre-pubertal population that deflazacort prevents excessive bone loss in comparison with methylprednisone maintenance. In one study, the use of deflazacort in kidney transplant patients was associated with a decrease in the loss of bone mineral density at the lumbar spine compared to prednisone [20]. The development of osteoporosis has been linked to negative calcium balance caused by the chronic administration of glucocorticoids at higher than physiological doses. Therefore, the fewer severe adverse effects on calcium balance observed with deflazacort treatment may indicate that it has a reduced adverse effect on bone mineral metabolism compared with prednisone. Several authors have supported this theory using a variety of methods, showing that deflazacort causes a decreased loss in bone mass or density compared to prednisone. Thus, it has been shown that deflazacort, although comparable to prednisone in the context of its anti-inflammatory activity, is significantly less harmful to cancellous bone than prednisone. Studies evaluating the ability of glucocorticoids to induce glycogen deposition have demonstrated that deflazacort is a potent enhancer of gluconeogenesis and hepatic glycogen synthesis, and it is approximately 10 times more effective when compared to prednisolone at equivalent doses. The metabolic side effects of corticosteroids occur due to chronic or long-term use. It is usually recommended to use a single dose of corticosteroids before or after the surgical removal of the impacted third molars. Therefore, it is not expected that the use of a single dose of corticosteroids will have significant negative effects on bone metabolism. The reason why we preferred deflazacort in this study is that its anti-inflammatory efficacy is higher than prednisolone and cortisol, and the duration of the anti-inflammatory action of deflazacort is longer than other glucocorticoids given in equivalent doses. It has been shown in the literature that deflazacort has fewer side effects than other corticosteroids, is more effective at similar doses, and lasts longer. In terms of studies in which deflazacort has been used in oral procedures, Anitua et al. used deflazacort prophylactically in dental implant surgery in individuals with oral lichen planus at a daily dose of 20 mg for 2 days preoperatively, 15 mg for 3 days postoperatively, and 7.5 mg for the next 3 days [35]. Konagala et al. used 30 mg of deflazacort as a single dose prior to root canal treatment to control post-endodontic pain [36]. De Vicente et al. used 60 mg of deflazacort on the day of surgery and 60 mg the following day in patients undergoing open maxillary sinus augmentation via the lateral approach [37]. A few studies in the literature show that the preferred dose and duration of deflazacort in dental procedures vary. Therefore, a single dose of 30 mg of deflazacort was preferred in this study.

Only one study was found in the literature that evaluated the effects of deflazacort on impacted lower third molar surgery (the study was in Spanish, and we found it in our detailed literature search), and in this study, Flores et al. compared the anti-inflammatory effects of deflazacort (given at 30 mg daily for 7 days) with betamethasone. They found that betamethasone was more effective in easing inflammation on the first postoperative day, and the effects of betamethasone and deflazacort on the duration of inflammation were similar [38]. Konagala et al. compared the analgesic effect of a single dose of 30 mg deflazacort, administered 1 h before root canal treatment, with piroxicam, dexamethasone, and a placebo after endodontic treatment [36]. They reported that pain levels were lower in the deflazacort group compared with the placebo group, and they were similar to the piroxicam and dexamethasone groups at 6, 12, and 24 h after surgery [36]. In this study, similarly to the study by Konagala et al., the pain scores were lower in the deflazacort group than in the control group at 6 and 12 h postoperatively [36]. There was no difference in swelling and trismus compared to the control group. These results suggest that the anti-inflammatory effect of a single dose of deflazacort is effective in the first 12 h postoperatively, depending on the half-life of the drug. The reason why there was no difference between the deflazacort group and the control group on postoperative day 2, the first day of trismus and swelling assessment, is thought to be due to the time-dependent decrease in the anti-inflammatory effect of deflazacort. The fact that pain, swelling, and trismus scores were similar in the control group on postoperative days 2 and 7 supports this idea. As there is no recommended dose of deflazacort in the literature for sub-embedded wisdom surgery, we used the study by Konagala et al. as a reference and preferred the single-dose 30 mg tablet form of the drug. If we had preferred a higher single dose or long-term use of the current dose, this could have changed the results [36].

The time and route of corticosteroid administration differ in impacted lower third molar surgery. They can be applied via the oral, intramuscular, or intramasseteric routes, and before or after the operation [39,40,41]. Dereci et al. applied immediate intramasseteric dexamethasone injections after impacted lower third molar surgery, and they indicated that immediate intramasseteric injection of dexamethasone was effective in reducing postoperative oedema [39]. Antonelli et al. evaluated the anti-inflammatory efficacy of a preoperative single 25 mg dose of prednisone tablets and reported significantly lower pain and oedema values compared to the control group [42]. Similarly, in the present study, patients who received preoperative 30 mg deflazacort tablets experienced less pain and swelling than the placebo group, even though the differences between facial oedema values were not statistically significant.

Miroshnychenko et al. reported a systemic review and meta-analysis about the use of corticosteroids for managing acute pain subsequent to surgical extraction of mandibular third molars. The results of this recent study demonstrated that patients receiving corticosteroids may experience a reduction in pain intensity compared with those receiving a placebo at 6 h and 24 h follow-ups [43]. In the same manner, we observed significantly lower pain levels in the deflazacort group at postoperative hours 6 and 12 compared to the placebo group.

Bakri et al. compared a postoperative single dose and three-day repetitive doses of 25 mg orally prescribed prednisolone and reported that a three-day administration of prednisolone was more effective in reducing post-extraction sequelae than a single-dose regimen [44]. In this study, we preferred a single preoperative dose of 30 mg deflazacort because its anti-inflammatory action lasts longer than other glucocorticoids. Considering that postoperative complications peak 48 to 72 h after the surgical procedure, significant differences in oedema and trismus values could have been obtained if patients had been administered repeated doses of deflazacort.

There are a few issues related to the limitations of the present study. In this clinical trial, the split-mouth study design was preferred to eliminate personal differences between individuals related to pain perception and the severity of inflammatory response. However, given that the teeth were not extracted in the same session in the split-mouth design, the pain threshold of patients may have changed based on their previous surgical experience, and tolerance to trismus may have developed. This phenomenon could explain why better mouth opening measurements were recorded in the placebo group. The postoperative administration of ibuprofen in both groups may have affected the assessment of the anti-inflammatory efficacy of deflazacort because NSAIDs may provide an additional benefit in reducing inflammation when given in conjunction with corticosteroids. Using tape measurement rather than more precise and sensitive methods such as magnetic resonance imaging, ultrasonography, or 3D face scanning for the assessment of facial swelling may be the reason for the lack of a significant difference in oedema values between the study and control groups.

## 5. Conclusions

Within the limitations of the study, a single preoperative dose of 30 mg deflazacort was found to be clinically effective in reducing pain and oedema after extraction of impacted lower third molars. Deflazacort may be an alternative treatment option to control postoperative sequelae associated with impacted third molar surgery. Further comparative clinical trials with other corticosteroids, which should include a larger sample size, should be conducted to evaluate the anti-inflammatory effects of deflazacort on postoperative complications after impacted lower third molar surgery and other oral surgical procedures.

## Figures and Tables

**Table 1:** Inclusion and exclusion criteria for the study.

Inclusion Criteria	Exclusion Criteria
* ASA 1, healthy * 18–40 years old * Bilateral impacted lower 3rd molars * Mesioangular (Angulation) * Class II and Class B (level of impaction)	* Systemic disease that interferes with surgical treatment * Smokers * Alcohol drinkers * Substance abusers * Pregnant and breastfeeding mothers * Patients with facial asymmetry * Patients currently receiving anti-inflammatory medication

**Table 2:** Demographic characteristic distribution.

Gender	Male (%)	12 (46.2)
	Female (%)	14 (53.8)
Age (year)	Min–Max (Median)	18–35 (22.5)
	Mean ± SD	23.31 ± 5.01

**Table 3:** Intergroup comparison of VAS scores.

	6th Hour		12th Hour		24th Hour		2nd Day		3rd Day		4th Day		5th Day		6th Day		7th Day	
	Mean ± SD	Median	Mean ± SD	Median	Mean ± SD	Median	Mean ± SD	Median	Mean ± SD	Median	Mean ± SD	Median	Mean ± SD	Median	Mean ± SD	Median	Mean ± SD	Median
Group 1	5.8 ± 2.1	6.00	4.81 ± 2.19	5.0	4.35 ± 3.02	5.0	3.92 ± 2.64	3.0	3.04 ± 2.72	2.5	1.96 ± 2.22	1.5	1.46 ± 1.90	0.5	1.04 ± 1.28	0.0	0.85 ± 1.54	0.0
Group 2	3.1 ± 1.6	3.00	3.27 ± 2.49	3.0	4.27 ± 2.71	5.0	4.04 ± 2.68	4.0	2.73 ± 2.41	2.0	1.77 ± 2.10	1.5	0.85 ± 1.26	0.0	0.65 ± 1.02	0.0	0.50 ± 0.81	0.0
*p*	^1^ 0.000 *		^1^ 0.025 *		^1^ 0.912		^1^ 0.861		^1^ 0.773		^1^ 0.832		^1^ 0.276		^1^ 0.290		^1^ 0.520	

^1^ Mann–Whitney *U* test. SD: standard deviation. Group 1: control; group 2: deflazacort. * *p* < 0.05.

**Table 4:** Intra-group comparison of the MMO values on postoperative days 2 and 7.

			n	Mean	SD	Median	Minimum	Maximum	*p*
Maximum mouth opening (%)	Day 2	Group 1	26	−31.93	15.25	−30.59	−60	−4.08	^1^ 0.701
		Group 2	26	−32.91	14.28	−34.52	−55.56	0.00	
	Day 7	Group 1	26	−13.17	11.75	−9.05	−43.75	0.00	^1^ 0.203
		Group 2	26	−17.22	11.99	−15.65	−41.67	−2.04	
			**n**	**Mean**	**SD**	**Median**	**Minimum**	**Maximum**	***p***
Maximum mouth opening (mm)	Pre-op	Group 1 and Group 2	26	44.5	5.13	45	31	55	^1^ 0.999
	Day 2	Group 1	26	30.15	7.17	30.00	16	47	^1^ 0.652
		Group 2	26	29.35	4.33	29.00	20	36	
	Day 7	Group 1	26	38.54	6.56	38.00	25	55	^1^ 0.478
		Group 2	26	36.58	5.36	38.00	28	48	

^1^ Mann–Whitney *U* test. SD: standard deviation; group 1: control group; group 2: deflazacort group.

**Table 5:** Intra-group comparison of facial measurements on postoperative days 2 and 7.

		Group 1 (%)		Group 2 (%)		Grup 1 (mm)		Grup 2 (mm)		*p*
		Mean ± SD	Median	Mean ± SD	Median	Mean ± SD	Median	Mean ± SD	Median	
Day 2 Distance		4.12 ± 2.13	3.67	3.07 ± 2.37	3.05	112.94 ± 9.22	112.83	111.68 ± 7.34	111.33	^1^ 0.089
Day 7 Distance		1.74 ± 1.80	1.12	1.44 ± 1.71	0.91	110.36 ± 8.93	109.67	109.95 ± 7.70	110.17	^1^ 0.349

^1^ Mann–Whitney *U* test. SD: standard deviation; group 1: control group; group 2: deflazacort group.

## Data Availability

Access to the dataset used in this study is available upon request. It is not publicly available as it contains information that could compromise the participants’ privacy.
